# The Anticonvulsant Activity of a Flavonoid-Rich Extract from Orange Juice Involves both NMDA and GABA-Benzodiazepine Receptor Complexes

**DOI:** 10.3390/molecules21091261

**Published:** 2016-09-21

**Authors:** Rita Citraro, Michele Navarra, Antonio Leo, Eugenio Donato Di Paola, Ermenegildo Santangelo, Pellegrino Lippiello, Rossana Aiello, Emilio Russo, Giovambattista De Sarro

**Affiliations:** 1 Department of Science of Health, School of Medicine and Surgery, University of Catanzaro, Catanzaro I-88100, Italy; citraro@unicz.it (R.C.); aleo@unicz.it (A.L.); donatodipaola@unicz.it (E.D.D.P.); santangelo@unicz.it (E.S.); aiellorossana@gmail.com (R.A.); erusso@unicz.it (E.R.); desarro@unicz.it (G.D.S.); 2 Department of Chemical, Biological, Pharmaceutical and Environmental Sciences, University of Messina, Messina I-98168, Italy; 3 Department of Pharmacy, University of Naples Federico II, Naples I-80131, Italy; plippiello@gmail.com

**Keywords:** orange, *Citrus sinensis*, flavonoids, audiogenic seizures, absence epilepsy, spike-wave discharges, natural products, complementary and alternative medicines, orange juice

## Abstract

The usage of dietary supplements and other natural products to treat neurological diseases has been growing over time, and accumulating evidence suggests that flavonoids possess anticonvulsant properties. The aim of this study was to examine the effects of a flavonoid-rich extract from orange juice (OJe) in some rodent models of epilepsy and to explore its possible mechanism of action. The genetically audiogenic seizures (AGS)-susceptible DBA/2 mouse, the pentylenetetrazole (PTZ)-induced seizures in ICR-CD1 mice and the WAG/Rij rat as a genetic model of absence epilepsy with comorbidity of depression were used. Our results demonstrate that OJe was able to exert anticonvulsant effects on AGS-sensible DBA/2 mice and to inhibit PTZ-induced tonic seizures, increasing their latency. Conversely, it did not have anti-absence effects on WAG/Rij rats. Our experimental findings suggest that the anti-convulsant effects of OJe are likely mediated by both an inhibition of NMDA receptors at the glycine-binding site and an agonistic activity on benzodiazepine-binding site at GABA_A_ receptors. This study provides evidences for the antiepileptic activity of OJe, and its results could be used as scientific basis for further researches aimed to develop novel complementary therapy for the treatment of epilepsy in a context of a multitarget pharmacological strategy.

## 1. Introduction

Epilepsy is one of the most common serious neurological disorders encountered in clinical practice. It is known that both GABA and glutamate receptors may play an important role in seizure initiation, maintenance and arrest [1]. Moreover, excessive activation of excitatory amino-acid receptors determines the generation of reactive oxygen (ROS) and reactive nitrogen (RNS) species that in turn can provoke seizure genesis and related cell death [2,3]. Thus, intervention with antioxidants could be a potential beneficial approach in the treatment of epilepsy [4]. Previous studies have suggested a protective role of antioxidants, such as ascorbic acid, vitamin E, α-tocopherol, curcumin, *trans*-resveratrol, melatonin and α-lipoic acid against seizures induced by different convulsive agents [5,6,7].

It is well known that flavonoids, plant secondary metabolites commonly found in the fruits and vegetables regularly consumed by humans, exert a broad spectrum of biological activities by both interaction with specific molecular targets and their antioxidant properties. Moreover, it is known that flavonoids are biologically active molecules in the central nervous system (CNS) [8] and that may act as ligands for benzodiazepine receptors [9]. Tea, as well as *Citrus* fruits and their juices, are the main food sources of flavonoids [10]. The health properties of *Citrus* flavonoids have been extensively studied, especially as regards their anticancer, cardiovascular and anti-inflammatory activity [11], however, to the best of our knowledge, the anticonvulsant potential of *Citrus* juices has not been investigated.

*Citrus sinensis* var. Tarocco, commonly known as “half-blood” orange is native to Italy and mainly cultivated in eastern Sicily. Its health properties have long been studied and were recently reviewed by Grosso et al. [12]. Very recently we studied the antioxidant activity of a flavonoid-rich extract from half-blood orange juice (OJe), demonstrating its capability to: (i) reduce the levels of both reactive oxygen species and membrane lipid peroxidation; (ii) improve mitochondrial functionality and (iii) prevent DNA-oxidative damage in A549 cells incubated with H_2_O_2_ [13]. We have also shown the chelating property of OJe and its ability to induce the antioxidant catalase, thus blocking iron oxidative-induced injury [14].

Based on this background, we have evaluated the potential anticonvulsant effects of an OJe on some animal models of seizures and epilepsy, investigating on its possible mechanism of action. Since hesperidin (HES) and narirutin (NRTN) were the flavonoids present in highest amounts in our extract, some anticonvulsant effects were also evaluated after their administration.

## 2. Results

### 2.1. OJe HES Mitigates Pentylenetetrazole (PTZ)-Induced Seizures

Following PTZ administration, all animals in the control group underwent both clonic and tonic seizures, and 75% died within 30 min (Table 1).

Administering OJe (40 mg/kg i.p.) 30 min before PTZ significantly (*p* < 0.01) induced an increase of latency (Table 2) and significantly suppressed tonic but not clonic seizures (Table 1A). Eighty mg/kg OJe significantly rise latency of tonus (*p* < 0.05), but not reduces the incidence of seizure phase (Table 1). On the contrary, both the dosage of 100 and 120 mg/kg significantly decrease the incidence of both clonus and tonus seizures (*p* < 0.01; Table 1A). Similarly, latency to both tonus and death episodes were significantly enhanced by OJe at the doses of 40, 80, 100 and 120 mg/kg (Table 2). Conversely, nor OJe at the dosage of 20 mg/kg neither HES or NRTN (40 and 80 mg/kg) significantly affected the incidence of seizures (Table 1A,B). Also OJe, HES or NRTN (all at 40 mg/kg concentration) administered 60 or 120 min before PTZ were ineffective. However, 80 mg/kg HES significantly enhanced the latency of tonus seizures and death (*p* < 0.05; Table 2A). Interestingly, oral treatment with both 20 and 40 mg/kg/day OJe for 5 consecutive days significantly (*p* < 0.05) inhibited both tonic seizures and death (*p* < 0.05 and *p* < 0.01, respectively; Table 1B) as well as the higher dose significantly increase their latency (*p* < 0.05; Table 2B). Similarly, oral administration of HES at 80 mg/kg/day concentration for 5 consecutive days significantly reduced the incidence of tonic seizures (*p* < 0.01; Table 1B) and increased their latency (*p* < 0.05; Table 2B), while the dose of 40 mg/kg/day appeared to reduce the incidence of tonic seizures and increase latency without reaching a significant level. On the contrary, NRTN (40 and 80 mg/kg/day) did not exert significant protection against PTZ-induced seizures (Table 1 and Table 2).

### 2.2. Effects of OJe, HES or NRTN in Audiogenic Seizure Prone DBA/2 Mice

**Figure 1:**
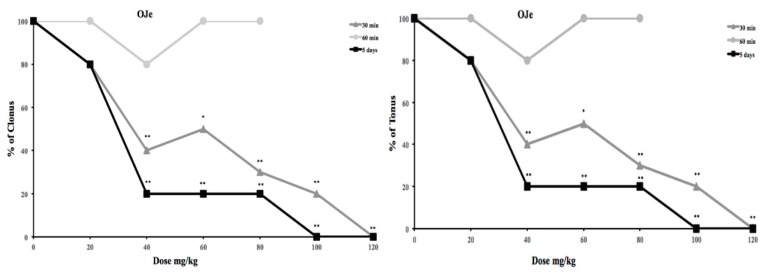
Effects of OJe against clonus and tonus in audiogenic seizure of DBA/2 mice.

OJe administration at dosage of 20 mg/kg (i.p.) 30 and 60 min before auditory stimulation did not influence the incidence of wild running and clonus of audiogenic seizures in DBA/2 mice. Instead, the administration of OJe at concentration of 40 mg/kg or higher 30 min before auditory stimulation significantly protected against tonus and clonus (*p* < 0.01; Figure 1), that were further reduced by the dosage of 100 and 120 mg/kg (*p* < 0.01).

Conversely, using the same experimental protocol, the administration of HES (up to 40 mg/kg) or NRTN (up to 60 mg/kg) with did not produce significant anticonvulsant effects against tonus and clonus (data not shown). Only a dose of HES of 120 mg/kg was able to significant protect against the clonic phase of audiogenic seizures, whereas NRTN was unable to protect against clonus at doses up to 120 mg/kg. The ED_50_ values of OJe, HES and NRTN against the tonus and clonus of audiogenic seizures were reported in Table 3.

Oral treatment for 5 consecutive days with OJe (20 mg/kg/day) before auditory stimulation did not exert marked anticonvulsant effects against the tonic and clonic phases of audiogenic seizures in DBA/2 mice. Of note, OJe administration at the doses of 40–120 mg/kg/day significantly reduced the incidence of both tonic and clonic seizures (*p* < 0.01; Figure 1 and Table 3). The same treatment with HES produced similar effects, but higher doses were necessary to produce similar antiseizure activity (Table 3), whereas NRTR was the weakest effective.

#### 2.2.1. Interactions between NMDA Antagonists (CPPene, d-Cycloserine, Felbamate) and OJe against Audiogenic Seizures in DBA/2 Mice

**Figure 2:**
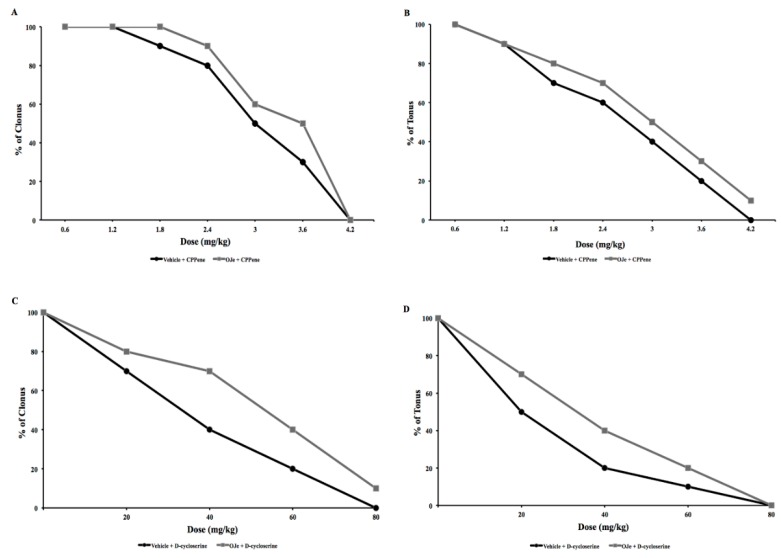
Influence of CPPene or d-cycloserine and their co-administration with OJe on the clonic (**A** and **C**) and the tonic (**B** and **D**) seizures in DBA/2 mice.

CPPene (0.6–4.2 mg/kg; i.p.) produced a dose-dependent protection against tonic and clonic phases of audiogenic seizures in DBA/2 mice when administered 45 min before auditory stimulation (Figure 2A,B) with an ED_50_ of 1.76 mg/kg for clonus and 0.79 mg/kg for tonus (Table 4).

**Figure 3:**
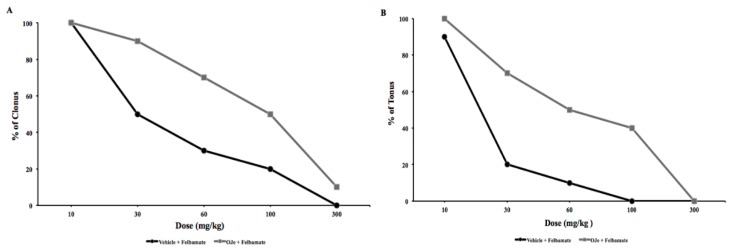
Influence of felbamate in presence or absence of OJe on both clonic (**A**) and tonic (**B**) seizures in DBA/2 mice.

In another group of experiments, we evaluated anticonvulsant effects of d-cycloserine (DCS), exposing the animals to auditory test. DCS (20–80 mg/kg; i.p.) administered 60 min before auditory testing induced a dose-dependent protection against the clonic and tonic phases of audiogenic seizure response in DBA/2 mice, with an ED_50_ of 27.6 mg/kg for clonus and 14.4 for tonus (Figure 2C,D and Table 4). Felbamate (30–300 mg/kg; i.p.) administered 45 min before auditory stimulation, dose-dependently reduced the severity of the audiogenic seizures in DBA/2 mice (Figure 3A,B). Felbamate antagonized audiogenic seizures with an ED_50_ value of 23.1 mg/kg for tonus and 48.8 mg/kg for clonus (Table 4).

In order to study the possible mechanism of action of OJe, the extract was administered at the not effective dose of 20 mg/kg in combination with DCS, felbamate or CPPene, 30 min before auditory stimulation. As shown in Figure 1 and Figure 2, OJe was able to produce a consistent shift to the right of the dose-response curves of CPPene, DCS and felbamate. Evidence that the maximum shift was observed when OJe was co-administered with felbamate and DCS, suggests that the decrease in anticonvulsant activity by OJe was prevalently mediated at the glycine site of NMDA receptor complex (Figure 2 and Figure 3). Accordingly, ED_50_ values were increased in co-administration protocols (Table 4).

#### 2.2.2. Interaction between NBQX and CFM-2, Two AMPA Receptor Antagonists and OJe

**Figure 4:**
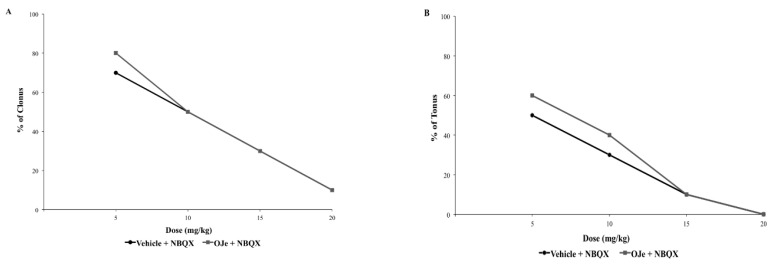

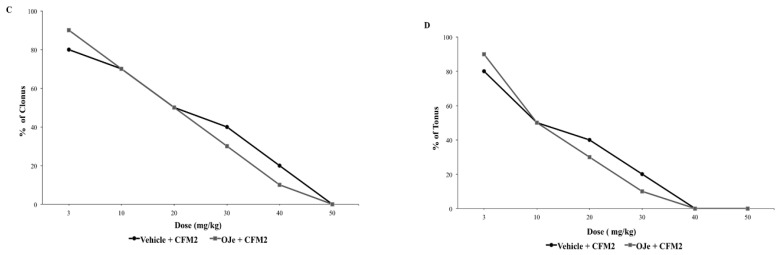
Influence of NBQX or CFM2 and co-administration with OJe on the clonic (**A** and **C**) and the tonic (**B** and **D**) seizures in DBA/2 mice.

NBQX (5–20 mg/kg; i.p.) administered 30 min before auditory stimulation, was able to suppress the severity of the audiogenic seizures in a dose-dependent manner (Figure 4A,B). Similarly, a pre-treatment of 30 min with CFM-2 (3–50 mg/kg; i.p.) produced a significant dose-dependent protection against tonic and clonic phases of the audiogenic seizures in DBA/2 mice (*p* < 0.05; Figure 4C,D). The ED_50_ values for NBQX and CFM-2 against clonic and tonic seizures are reported in Table 4. Co-administration of OJe (20 mg/kg; i.p.) with the two competitive AMPA receptor antagonist 30 min before auditory stimulation was unable to produce a consistent shift of the dose-response curves for both drugs (Figure 4 and Table 4) The only case of significant increase (*p* < 0.05) of ED_50_ value for clonus was observed when OJe was co-administered with CFM-2.

#### 2.2.3. Treatment with Flumazenil

**Figure 5:**
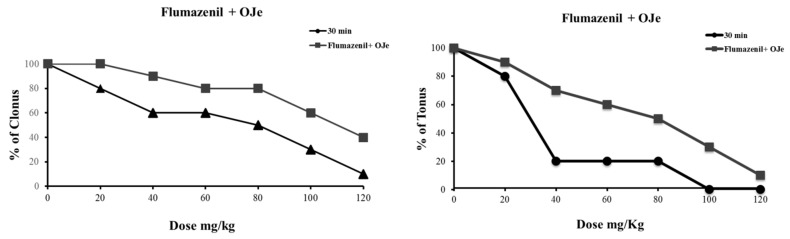
Influence of flumazenil together or not with OJe on both clonic and tonic seizures in DBA/2 mice.

To ascertain the possible involvement of GABA-benzodiazepine receptor complex in the antiseizure activity of OJe, the latter was administered 15 min before flumazenil. As shown in Figure 5, the anticonvulsant effect of OJe (40 mg/kg; i.p.) was reduced by a treatment with 2.5 mg/kg flumazenil. Flumazenil administered i.p. 15 min before HES or NRTN antagonized the modest pharmacological effects of these flavonoids (data not shown).

### 2.3. Effects of OJe on Absence Seizures in WAG/Rij Rats

At 6 months of age, all WAG/Rij rats exhibited spontaneously occurring SWDs on EEGs; the mean number of SWDs (nSWDs) for a 30 min epoch was 5.82 ± 0.84 min seizures with a mean total duration (dSWDs) of 12.23 ± 5.34 s. The i.p. administration of OJe (20 and 40 mg/kg), HSE or NRTN (40 and 80 mg/kg) did not modify the number and duration of SWDs in comparison to control group (data not shown).

## 3. Discussion

In this study, for the first time, we report the anticonvulsant effects of a flavonoid-rich extract from *Citrus sinensis* juice in some experimental models of epilepsy. Our results support the effects of OJe in the CNS, showing that more than a single mechanism of action might contribute to its anti-seizure properties. The main mechanisms involved in the anticonvulsant activity exerted by OJe seems to be linked to its effects on both GABA_A_ and NMDA receptors.

It is well known that several natural products widely used in traditional, folk and alternative medicine exert a broad spectrum of biological activities, so many plant extracts are currently used for the prevention or treatment of certain diseases. Very often their pharmacological activity is due to the presence of flavonoids, plant secondary metabolites commonly found in the fruits and vegetables regularly consumed by humans. In this line, we have recently shown the anti-tumor effects of the flavonoid fraction of *Citrus reticulata* (mandarin) juice [15], as well as we demonstrated that the anti-cancer activity of the *Citrus bergamia* (bergamot) juice (BJ) exerted both in vitro [16,17] and in vivo [18] is related to its flavonoids [19]. Interestingly, evidence that a flavonoid-rich extract from bergamot juice (BJe) is able to exert antioxidant and anti-inflammatory activity both in vitro [20,21] and in animal models [22,23] increases the potential of the *Citrus* juice as a tool for pharmacological intervention in some pathologies [24,25]. *Citrus sinensis* var. Tarocco, commonly known as ”half-blood“ orange is native to Italy and mainly cultivated in eastern Sicily. Its health properties were studied and are recently reviewed by Grosso et al. [12]. The OJe employed in this experimental research has demonstrated antioxidant properties, realized by different complementary routes via scavenging free radicals, chelating metal ions and boosting the cellular antioxidant defense [13,14].

Data from the present study demonstrate the anticonvulsant activity of OJe in PTZ-induced seizures in CD-1 mice and in audiogenic seizures in DBA/2 mice, but not against absence seizures in WAG/Rij rats. However, OJe not always induced a dose-dependent response, and we can’t exclude that other effects might appear at higher dosages. Since OJe is a pool of flavonoids, it is likely that more than a single bioactive molecules present in the extract could contribute to the observed effects. Accordingly, hesperidin, the major component of our extract, was found to be effective against PTZ-kindling at dosages (100–200 mg/kg) much higher than the one used in our experiments (about 10 mg/kg) [26]. In addition, OJe appeared more potent and effective than HES and NRTN, suggesting that other components are involved in the antiseizure effects. This was supported by the evidence that a much higher dose of HES or NRTN given alone or in combination is needed to obtain comparable pharmacological results to those observed using the OJe. These findings strengthen the hypothesis that the complex mixtures of phytochemicals present in an extract could be more effective than their individual constituents, enhancing each other’s pharmacological activity. Moreover, a phytocomplex has the advantage that the individual active substances are present in a much lower concentration than that would be required to achieve the same effectiveness with a single active principle, with possible impact on the safety of the natural drug. Finally, the presence of numerous molecules that simultaneously can act on different targets and by diverse mechanisms of action may enhance the potential of phytocomplexes in a context of a multitarget pharmacological strategy.

GABA and glutamate are the major neurotransmitters in the brain, and are involved in the pathophysiology of epilepsy [27]. In order to explore the mechanism through which OJe exerts its antiseizure activity, first we used the flumazenil, a benzodiazepine receptor antagonist [28]. Evidence that this drug was able to antagonize most of OJe effects indicate that the *Citrus* extract act as agonist of the GABA-benzodiazepine receptor complex. Also HES and NRTN appears to act in this manner.

The excitatory neurotransmitter glutamate has been implicated in early changes that lead to the initiation of hyperactivity, but also to the amplification and spread of the excitatory hyperactivity acting through two main families of receptors, the ionotropic and metabotropic glutamate receptors [29]. Both types of receptors have been implicated in the etiology of different seizures types [30,31]. In this light, in the DBA/2 mice, a genetic animal model of audiogenic seizures, we have studied OJe activity on glutamate ionotropic receptors by combination of our extract with various antagonists of these receptors. OJe at a concentration of 40 mg/kg administrated 30 min before auditory stimulation possesses anticonvulsant properties in DBA/2 mice with significant protection only against tonus. At least 80 mg/kg were necessary to protect against clonus. Moreover, oral treatment for 5 consecutive days protected DBA/2 mice from sound-induced tonic extension. Then, we have used the not effective dose of 20 mg/kg in combination with glutamate receptor antagonists. It is known that CPPene, felbamate, DCS, NBQX and CFM-2 are effective anticonvulsants in DBA/2 mice [32,33,34]. Among these drugs, OJe interacted with both DCS and felbamate, which are known to bind at the glycine modulatory site on the NMDA receptor complex [35]. At odds, OJe interacts very slightly with CPPene, acting on the glutamate binding site on the NMDA receptors, and not at all with NBQX or CFM-2, two competitively and non-competitively blockers of AMPA receptors, respectively. These data suggest that the anticonvulsant activity of OJe was also mediated by the interaction with the glycine site of NMDA receptor complex.

Taken together, our results demonstrate that the flavonoid-rich extract from orange juice employed in this study possesses antiepileptic effects in PTZ-induced seizures and in AGS-sensible DBA/2 mice, which are very likely mediated by both the inhibition of NMDA receptors at the glycine-binding site and the agonistic activity on benzodiazepine-binding site at GABA_A_ receptors. However, other mechanisms of action contributing to the effects exerted by OJe cannot be excluded, and further studies will be necessary to explore the detailed mechanism of OJe action at the CNS.

This study provides evidences for the antiepileptic activity of OJe, and its results could be used as scientific basis for further researches aimed to develop novel complementary therapy for the treatment of epilepsy in a context of a multitarget pharmacological strategy.

## 4. Materials and Methods

### 4.1. Animals

Male DBA/2 mice (3 weeks of age), CD-1 mice (6 weeks of age) and WAG/Rij rats (6–7 months old, 250–300 g) were purchased from Harlan Italy Srl (Correzzana, Milan, Italy). Animals were housed in groups under stable conditions of humidity (60% ± 5%) and temperature (21 ± 2 °C), with a reversed light/dark (12/12 h) cycle (light on at 19.00) with free access to standard laboratory chow and tap water until the time of experiments. Procedures involving animals and their care were conducted in conformity with the international and national laws and policies (EU Directive 2010/63/EU for animal experiments, ARRIVE guidelines and the Basel Declaration including the 3R concept). All efforts were made to minimize animal suffering and to use only the number of animals necessary to produce reliable scientific data.

### 4.2. Drugs

**Figure 6:**
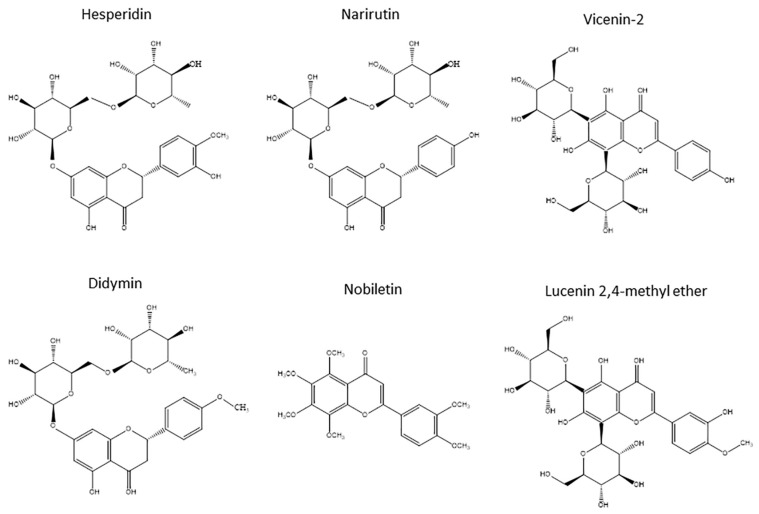
Chemical structures of the main flavonoids in OJe.

The OJe was provided by the company “Agrumaria Corleone” (Palermo, Italy) that used fruits of *Citrus sinensis* (L.) Osbeck (sweet orange) var. Tarocco coming from crops grown in the south-eastern part of Sicily (Italy). The extract was produced in its liquid form by passing the fresh orange juice through columns equipped with adsorbent resins that retain flavonoids. The latter were then eluted with NaOH and immediately passed through cationic resins, thus obtaining the biomolecules in their acid form. Finally, the extract was collected, filtered, centrifuged, transformed into a powder by spray drying and then stored at −20 °C. Immediately prior to use, it was defrosted, diluted in saline solution until the desired concentration and administered orally by gavage or intraperitoneally, depending on the test. Qualitative and quantitative composition of OJe was previous reported [13]. The flavanones hesperidin and narirutin were the flavonoids present in highest amounts (232 and 90 mg/g, respectively), followed by the flavone *C*-glucosides vicenin-2 and lucenin-2 methyl ether (43 and 22 mg/g of dried extract, respectively). Didymin and nobiletin were the flavanone *O*-glycosides and the polymetoxyflavone present in quite large quantities, respectively (15 mg/g of dried extract for both). Their chemical structures are shown in Figure 6.

CPPene (3-((F)-2-carboxypiperazin-4-yl)-1-phosphonic acid) was supplied by Novartis Pharmaceutical Development (Basel, Switzerland) and d-Cycloserine (d-4-amino-3-isoxazolidone, DCS) was purchased from Sigma (Milan, Italy). Felbamate was supplied by Schering-Plough (Milan, Italy), 2,3-dihydroxy-6-nitro-7-sulphamoyl-benzo(F)quinoxoline (NBQX) by Novo Nordisk (Malov, Denmark), CFM-2, (1-(4-aminophenyl)-3,5-dihydro-7,8-dimethoxy-4*H*-2,3-benzodiazepin-4-one) was synthesized in the A. Chimirri laboratories (University of Messina, Italy). Flumazenil (ethyl-8-fluoro-5,6-dihydro-5-methyl-6-oxo-4*H*-imidazo[1,5-a][1,4]benzodiazepine-3-carboxylate) was obtained from Hoffmann-LaRoche (Basel, Switzerland). Hesperidin (HES) and narirutin (NRTN) were purchased from Sigma (Milan, Italy). HES was suspended in 0.5% *w*/*v* sodium carboxymethylcellulose (CMC), while NRTN was dissolved in sterile saline; both flavonoids were administered orally (p.o.) by gavage or intraperitoneally (i.p.), depending on the test. HES and NAR were administered at the same times of OJe before some convulsant tests. All the other compounds were given i.p. (0.1 mL/10 g of mouse’s body weight). CPPene and DCS were dissolved in sterile saline and given i.p. as freshly prepared solution. Felbamate and CFM-2 were administered as a freshly prepared solution in 50% dimethylsulphoxide (DMSO) and 50% sterile saline (0.9% NaCl). NBQX was dissolved in a minimum quantity of NaOH 1 N. The final volume was made up with sodium phosphate buffer (67 mM). When necessary, the pH was adjusted to 7.3–7.4 by adding HC1 0.2 N.

### 4.3. Pentylenetetrazole (PTZ)-Induced Seizures in CD-1 Mice

In order to investigate the effects of OJe treatment on PTZ-induced seizures, we used two different administration protocols.

#### 4.3.1. Experiment 1 (Acute Treatment)

The effect of acute systemic OJe, HES or NRTN administration on PTZ-induced seizures was investigated in CD-1 mice, that received OJe, HES or NRTN (20, 40, 80, 100 or 120 mg/kg i.p. *n* = 8 mice per dose) or its vehicle (sterile saline solution 0.9% NaCl i.p.), 30, 60 or 120 min before the injection of PTZ (65 mg/kg; i.p.) [36]. Animals were placed in a 30 × 30 × 30 cm Plexiglas box and observed for 30 min to evaluate the occurrence of clonic and tonic seizure and their latency; a threshold convulsion has been considered as an episode of clonic spasms lasting for at least 5 s [28]. Absence of this threshold convulsion over 30 min indicated that the animal was protected from the convulsant-induced seizures [37].

#### 4.3.2. Experiment 2 (Subchronic Treatment)

OJe, HES, NRTN or vehicle were orally administered for 5 consecutive days (20 or 40 mg/kg/day; *n* = 10 mice per dose) and the last administration was 30 min before PTZ (dose of 60 mg/kg) injection as previously described [36,37]. The animals were evaluated for the appearance of behavioral seizures, as described above.

### 4.4. Audiogenic Seizures in DBA/2 Mice

Experimental groups, consisting of 10 animals, were assigned according to a randomized schedule, and each mouse was used only once. Control animals were always tested on the same day with respective experimental groups, as previously described [37].

For acute treatment (Experiment 1), DBA/2 mice were exposed to auditory stimulation 30, 45, 60 and 120 min following intraperitoneal (i.p.) administration of OJe, HES or NRTN at the doses of 20, 40, 60, 80, 100 and 120 mg/kg (*n* = 10 mice per dose). Each mouse was placed under a hemispheric Perspex dome (diameter 58 cm) and 1 min was allowed for habituation and assessment of locomotor activity. Auditory stimulation (12–16 kHz, 109 dB) was applied for 1 min or until tonic extension occurred. The seizure response was assessed using the following scale: 0 = no response, 1 = wild running, 2 = clonus, 3 = tonus, 4 = respiratory arrest, as previously reported [38]. The maximum response was recorded for each animal.

For subchronic treatment (Experiment 2), DBA/2 mice were divided into four groups and orally pretreated, for 5 days, with OJe, HES or NRTN (20, 40, 60, 80, 100 and 120 mg/kg/day; *n* = 10 mice per dose) before auditory testing, as above described.

#### 4.4.1. Administration of NMDA and AMPA Receptor Antagonists with OJe in DBA/2 Mice

For NMDA and AMPA antagonist receptors testing, DBA/2 mice were exposed to auditory stimulation 45 min following administration of vehicle, CPPene (at least *n* = 60 mice for each group) or felbamate (*n* = 50 mice for each group), 60 min following injection of DCS (*n* = 50 mice for each group) and 30 min following injection of NBQX (*n* = 40 mice for each group). or CFM-2 (*n* = 60 mice for each group). For co-administration, DBA/2 mice were pretreated with OJe (20 mg/kg, i.p.), 15 min before CPPene or felbamate administration, and 30 min before DCS, NBQX or CFM-2 administration. Auditory stimulation and seizure evaluation were performed as above described.

#### 4.4.2. Co-Administration of Flumazenil with OJe in DBA/2 Mice

DBA/2 mice were administered i.p. with OJe, HES or NRTN (10–80 mg/kg *n* = 10 mice per dose) 15 min before flumazenil (2.5mg/kg i.p.), and auditory test was performed 30 min later. The dose of flumazenil, used in the present experiments was chosen according to our previous article because it does not worsen audiogenic seizures when administered alone. Total number of mice that developed seizures was tallied at each dose [28].

### 4.5. Experiments in WAG/Rij Rats

WAG/Rij rats of about 6 months of age and a body weight of approximately 280 g were chronically implanted, under anesthesia obtained by administration of a mixture of tiletamine/zolazepam (1:1; Zoletil 100^®^; 50 mg/kg i.p.; VIRBAC Srl, Milan, Italy), using a Kopf stereotaxic instrument, with five cortical electrodes for EEG recordings, as previously described [39]. All animals were allowed to at least 1 week of recovery and handled twice a day. In order to habituate the animals to the recording conditions, rats were connected to the recording cables, for at least 3 days before the experiments. The animals were connected to a multichannel amplifier (Stellate Harmonie Electroencephalograph; Montreal, QC, Canada) by a flexible recording cable and an electric swivel, fixed above the cages, permitting free movements for the animals [40]. WAG/Rij rats were intraperitoneally (i.p.) administered with different doses of OJe (20 and 40 mg/kg). Separate groups of rats (*n* = 6 for each dose) were used to determine the effects of vehicle (saline) and drug on the number and duration of SWDs. Every EEG recording session lasted 5 h:1 h baseline without injection, and 4 h after the i.p. administration of OJe, HES or NRTN or vehicle [39]. All EEG signals were amplified and conditioned by analog filters (filtering: below 1 Hz and above 30 Hz at 6 dB/octave) and subjected to an analog-to-digital conversion with a sampling rate of 300 Hz. The quantification of absence seizures was based on the number and the duration of electroencephalogram spike-wave discharges (SWDs), as previously described [41].

### 4.6. Statistical Analysis

All statistical procedures were performed using SPSS 15.0. software (Windows version 15.0, SPSS Inc., Chicago, IL, USA). In DBA/2 mice, statistical comparisons between control and drug-treated groups, were made using Fisher’s exact probability test (incidence of the seizure phases). The percentage incidence of each phase of the audiogenic seizure was determined for each dose of compound administered, and dose-response curves were fitted using linear regression analysis of percentage response. ED_50_ values (±95% confidence limits) for each compound and each phase of seizure response were estimated using the method of Litchfield and Wilcoxon (1949) [42]. The relative anticonvulsant activities were determined by comparison of respective ED_50_ values [43]. Means ± SEM. were calculated for all relevant measures. Seizure severity scores and latencies were compared between groups using a Kruskall-Wallis nonparametric analysis of variance (ANOVA) followed by a Mann-Whitney U-test. For experiments in WAG/Rij rats, EEG recordings were subdivided into 30 min epochs, and the duration and number of SWDs were treated separately for every epoch. These values were averaged and data obtained were expressed as mean ± SEM for each dose group. Treated animals were compared by one-way ANOVA with treatment as the only variable, followed by a Bonferroni’s post hoc test. All tests were two-sided, with *p* < 0.05 being considered significant.

## Figures and Tables

**Table:** **A**

Treatment (mg/kg; i.p.)		Time(min)	Seizure Phase (%)			Number of Mice
			Clonus	Tonus	Death	
Vehicle	Saline	30	100	100	75	8
OJe	20	30	100	87.5	75	8
	40	30	75	37.5 **	37.5 **	8
	80	30	75	50 *	75	8
	100	30	50 *	25 **	25 **	8
	120	30	25 **	0 **	0 **	8
	40	60	100	75	75	8
	40	120	100	100	100	8
HES	40	30	100	87.5	75	8
	80	30	100	50 *	50 *	8
	100	30	62.5	25 **	25 **	8
	120	30	25 **	12.5 **	0 **	8
	40	60	100	100	87.5	8
	40	120	100	100	87.5	8
NRTN	40	30	100	100	75	8
	80	30	100	75	75	8
	100	30	87.5	50 *	50 *	8
	120	30	62.5	25 **	37.5 **	8
	40	60	100	100	100	8
	40	120	100	100	100	8
HES + NRTN	120 + 120	30	37.5 **	12.5 **	12.5 **	8

**Table:** **B**

Treatment (mg/kg; os)		Time (Days)	Seizure Phase (%)			Number of Mice
			Clonus	Tonus	Death	
Vehicle	Saline	5	100	100	100	10
OJe	20	5	100	60 *	40 **	10
	40	5	80	20 **	20 **	10
HES	40	5	100	75	50 *	8
	80	5	90	40 **	30 **	10
NRTN	40	5	100	100	80	10
	80	5	100	100	75	8

**Table:** **A**

Treatment (mg/kg; i.p.)		Latency (s)		
		Clonus	Tonus	Death
Vehicle	Saline	164 (152–177)	681 (618–750)	706 (586–851)
OJe	20	174 (158–192)	712 (584–868)	776 (645–934)
	40	194 (176–214)	852 (695–1044) *	918 (746–1130) *
	80	211 (192–230)	868 (684–1102) *	909 (684–1208) *
HES	40	176 (156–198)	734 (592–910)	804 (672–962)
	80	184 (160–211.6)	845 (683–1110) *	878 (724–1064.8) *
NRTN	40	168 (150–201)	756 (564–900)	801 (644–966)
	80	179 (155–206.7)	784 (598–1027.8)	834 (696–999.4)

**Table:** **B**

Treatment (mg/kg; 5 Days; os)		Latency (s)		
		Clonus	Tonus	Death
Vehicle	Saline	158 (140–178)	664 (598–737)	712 (604–839)
OJe	20	160 (140–180)	670 (494–697)	788 (602–946)
	40	224 (198–253)	928 (715–1204) *	998 (776–1284) *
HES	40	168 (150–201)	756 (564–900)	801 (644–966)
	80	218 (200–248)	899 (709–1198) *	995 (788–1226) *
NRTN	40	155 (146–198)	744 (526–897)	798 (625–978)
	80	196 (150–201)	767 (602–916)	802 (627–974)

**Table 3:** ED_50_ values (±95% confidence limits) of the OJe, HES and NRTN on audiogenic seizures in DBA/2 mice. All data are expressed in mg/kg and were calculated according to the method of Litchfield and Wilcoxon (1949). The significant changes were statistically evaluated according to Lichtfield and Wilcoxon. ** *p* < 0.01.

Treatment	Time (min)	ED_50_ Values	
		Clonus	Tonus
OJe	30	71.89 (56.75–91.08) **	36.34 (25.49–51.82) **
HES	30	112.05 (81.85–153.40)	57.54 (47.33–69.95)
NRTN	30	>120	66.65 (48.49–91.22)

**Table 4:** ED_50_ values (±95% confidence limits) of the OJe co-administrated with NMDA antagonists on audiogenic seizures in DBA/2 mice. All data are expressed in mg/kg and were calculated according to the method of Litchfield and Wilcoxon (1949). The significant changes were statistically evaluated according to Lichtfield and Wilcoxon. * *p* < 0.05; ** *p* < 0.01.

Treatment			ED_50_ Values	
			Clonus	Tonus
CPPene	plus	Saline	1.76 (1.21–2.26)	0.79 (0.44–1.43)
	plus	OJe	2.69 (2.30–3.15) **	2.61 (2.04–3.33) **
d-cycloserine	plus	Saline	27.6 (17.7–43.2)	14.4 (7.8–26.5)
	plus	OJe	58.7 (37.4–92.3) **	28.3 (20.1–39.8) **
Felbamate	plus	Saline	48.8 (35.4–67.2)	23.1 (12.1–44.0)
	plus	OJe	105.6 (64.9–171.7) **	65.9 (38.5–112.8) **
NBQX	plus	Saline	4.9 (3.2–7.5)	2.2 (1.4–3.6)
	plus	OJe	4.1 (2.6–6.49)	2.8 (2.05–3.73)
CFM–2	plus	Saline	10.04 (11.3–13.1)	9.42 (7.34–12.09)
	plus	OJe	15.9 (11.3–22.46) *	10.78 (7.59–15.30)
